# Unraveling the mechanisms of fruit abscission in *Morus laevigata* through multi-omics approaches

**DOI:** 10.3389/fpls.2025.1605312

**Published:** 2025-09-22

**Authors:** Jiahu Yang, Sha Li, Zhennan Li, Yahui Xuan, Jiamei He, Mingju Ruan, Yuming Feng, Zhenyang Tao, Xiaoru Kang, Zhengang Li

**Affiliations:** ^1^ Sericulture and Apiculture Research Institute, Yunnan Academy of Agricultural Sciences, Mengzi, Yunnan, China; ^2^ State Key Laboratory of Resource Insects, Southwest University, Beibei, Chongqing,, China

**Keywords:** *Morus laevigata*, fruit abscission, plant carbohydrates, plant hormone, auxin, abscisic acid

## Abstract

**Introduction:**

*Morus laevigata* (long-fruited mulberry) is rich in active components and possesses significant nutritive value. The fruitlet stage represents a critical period for fruit abscission, and elucidating the underlying biological mechanisms of this process can provide a theoretical foundation for breeding more stable and abscission resistant cultivars.

**Methods:**

Fruit peduncles at the fruit set stage (April to May) were selected as experimental materials, including both abscising and non-abscising fruits. Morphological analysis of the peduncle abscission zone was conducted to examine structural differences. Additionally, transcriptomic and metabolomic analyses were performed to investigate gene expression and metabolite changes associated with fruit abscission.

**Results:**

Morphological analysis of the peduncle abscission zone in abscising fruits revealed enlarged intercellular spaces and disorganized cell arrangements. Transcriptomic and metabolomic analyses indicated that genes and metabolites related to fruit abscission were primarily involved in plant hormone signal transduction, and starch and sucrose metabolism pathways. Auxin and abscisic acid were identified as key regulators, modulating the expression of cell wall-degrading enzymes, which facilitated cell wall loosening and degradation, ultimately leading to fruit abscission. Furthermore, alterations in energy metabolism were found to play a pivotal role in this process.

**Conclusion:**

These findings contribute to a deeper understanding of the physiological mechanisms underlying fruit development and abscission, offering valuable insights into mulberry breeding and the sustainable advancement of modern agriculture.

## 1 Introduction

*Morus laevigata*, commonly known as the long-fruited mulberry, belongs to the genus *Morus* L. within the family Moraceae. It is widely cultivated in various regions worldwide, particularly in the subtropical areas of Asia (Lee and Hwang, 2017). In addition to its high nutritional value, mulberry plays a significant role in traditional medicine (Eisenbrand and Tang, 2010), with numerous studies reporting that its active components confer various health benefits (Sarkhel et al., 2020; Yuan et al., 2017). Mulberry produces a multiple fruit, which is formed from the ovaries of many flowers borne on a single inflorescence. Each small drupelet within the fruit originates from an individual flower, and the entire structure matures into a fleshy, compound fruit. The peduncle supports the inflorescence and later the developing multiple fruit, playing a vital role in nutrient transport and fruit retention (Ozturk et al., 2023). During mulberry cultivation, fruit abscission is common and severe that significantly affects fruit yield and quality, thereby reducing economic benefits. Therefore, effective regulation of fruit abscission is crucial for improving productivity and profitability in mulberry production.

Fruit abscission is a vital physiological process that enables plants to shed mature, damaged, or unnecessary organs, thereby supporting reproductive success and seed dispersal. This process is tightly regulated and involves the differentiation and activation of a specialized cell layer known as the abscission zone (AZ) (Shi et al., 2023; van Nocker, 2009). Among the key regulators, plant hormones play a central role in modulating AZ development and activity. For instance, ethylene has been reported to induce fruit abscission (Kenea, 2021), while auxin can inhibit this process in various plant species (Li and Su, 2024). In sweet cherry, abscisic acid (ABA) has been shown to accumulate significantly during fruit abscission (Qiu et al., 2021). Additionally, in mulberry, ABA-related transcription factors MaABF1 and MaABI5 have been shown to antagonistically regulate the expression of *MaJOINTLESS*, a key gene involved in AZ development, further highlighting the pivotal role of ABA signaling in fruit abscission (Deng et al., 2023). Overall, fruit abscission is governed by a complex hormonal network, in which the crosstalk among ethylene, auxin, ABA, and other phytohormones plays a pivotal role in coordinating the timing and progression of cell separation processes in diverse crop species, such as apple (Sriskantharajah et al., 2021), tomato (Dong et al., 2024), and cotton (Zhao et al., 2024b). Conversely, hormonal imbalances can disrupt these regulatory signals, leading to fruit drop at various developmental stages, including the young fruitlet stage (Kumar and Kumar, 2016). Activation of the AZ is also closely associated with cell wall–modifying enzymes, including cellulases, polygalacturonases (PGs), and pectin methylesterases (PMEs). These enzymes degrade components key structural components of the primary cell wall and the middle lamella, thereby weakening cell-to-cell adhesion and facilitating organ detachment (Li and Su, 2024). The essential roles of cellulases in abscission are well established, with cellulase-encoding genes identified in various plant species, such as *GH9* family genes in soybean (Zhan et al., 2025), citrus (Merelo et al., 2017), and mulberry (Deng et al., 2024). Enzymes such as pectate lyase have also been implicated in the abscission processes across multiple species including *Arabidopsis thaliana* (Niederhuth et al., 2013), soybean (Tucker et al., 2007), banana (Mbéguié et al., 2009), and rose (Singh et al., 2011). The coordinated activity of these enzymes, particularly cellulases and pectinases, leads to the breakdown of cellulose and pectin within the AZ and is considered one of the primary drivers of organ detachment (Bonghi et al., 1992; Roongsattham et al., 2016; Taylor and Whitelaw, 2001). In addition to hormonal and enzymatic regulation, carbohydrate metabolism also contributes to the regulation of fruit abscission (Zhu et al., 2011b). Carbohydrate depletion caused by impaired photosynthesis, resulting from either defoliation or shading treatment, has been shown to induce fruit abscission in various species (Mehouachi et al., 1995; Fei et al., 2025).

Recent advances in metabolomics and transcriptomics have enabled the systematic analysis of gene expression patterns and metabolic profiles associated with fruit abscission. The AZ represents a metabolically dynamic region involving complex networks of hormones, carbohydrates, and secondary metabolites (Tranbarger and Tadeo, 2025). In blue honeysuckle (*Lonicera caerulea*), non-targeted metabolomics revealed that differentially accumulated metabolites (DAMs) between easy- and hard-abscission cultivars were significantly enriched in pathways such as phenylpropanoid biosynthesis, starch and sucrose metabolism, and amino sugar metabolism, along with hormone biosynthesis routes like tryptophan and carotenoid metabolism in the fruit AZ (Chen et al., 2023a). Transcriptomic studies have identified multiple genes associated with AZ development and formation (van Nocker, 2009; Zhao et al., 2024a; Pautot et al., 2025). MACROCALYX and JOINTLESS, both MADS-box transcription factors, have been shown to regulate the expression of genes related to phytohormone signaling, cell wall modification, and key developmental transcription factors specifically in the tomato pedicel AZ (Nakano et al., 2012). In contrast, mutations in *JOINTLESS* impairs AZ development, resulting in the failure of organ separation at the normal abscission site (Huerga-Fernández et al., 2024). In mulberry, transcriptomic analysis showed that enrichment of the DEGs between easy-to-drop and less abscission-prone fruit stalks was significantly associated with pathways including flavonoid biosynthesis, the citric acid cycle, phytohormone signaling, and amino acid biosynthesis (Deng et al., 2023). Similarly, DEGs identified between abscised and retained young fruits were enriched in the same or related metabolic and signaling pathways (Xuan et al., 2023).

Despite the progress in current research, the molecular mechanisms underlying fruit abscission in mulberry remain largely unexplored. In this study, we integrated metabolomic and transcriptomic analyses to compare peduncles from abscising and non-abscising fruits of two mulberry varieties with contrasting abscission rates. The integration of these datasets offers novel insights into the coordinated regulation of gene expression and metabolite accumulation in the mulberry AZ, providing a valuable foundation for understanding the genetic and biochemical basis of fruit shedding in this species.

## 2 Materials and methods

### 2.1 Plant materials

This study utilized fruit peduncles as experimental materials, which were collected on March 15, 2023, from the Mulberry Germplasm Resource Garden of Yunnan Province. Two *M. laevigata* varieties with contrasting fruit abscission rates were selected: ‘Peach Fairy,’ characterized by a low fruit abscission rate, and ‘Pink Lady,’ exhibiting a high fruit abscission rate. Three healthy, disease-free three-year-old trees of each variety were randomly selected, with each tree considered a biological replicate. Fifteen days after flowering, three types of samples were collected: non-abscising fruits from ‘Peach Fairy’ (PF), as well as non-abscising (PL-N) and abscising fruits from ‘Pink Lady’ (PL-A). Non-abscising fruits were carefully selected based on their firmness and strong attachment to the branch, showing no signs of looseness or shriveling. Fruits considered to be abscising were identified by their ease of detachment, as they fell off with slight touch and had a shriveled appearance, indicating imminent abscission. For each sample, fruits were harvested from different orientations of the same tree, and their peduncles were excised and pooled. Immediately after collection, the peduncles were flash-frozen in liquid nitrogen, transferred into test tubes, and transported to the laboratory, where they were stored at -80 °C for further analysis.

The sampling time corresponds to the peak period of fruit abscission, as determined by monitoring abscission rates. Fruit drop began approximately 10 days after flowering and reached its highest level around the sampling time. To assess abscission rates, three branches were selected on each tree, and the initial fruit number was recorded. Subsequently, the number of fruits remaining on these branches was counted at different time points to calculate the rate of fruit drop (**Supplementary Table 1**).

### 2.2 Anatomical examination of fruit peduncles

Fresh fruit peduncles were collected and fixed in a standard FAA (formalin, acetic acid, and 70% ethanol) solution for 24 h. Following fixation, the tissues were processed for paraffin embedding, with longitudinal and transverse sections cut along the longitudinal axis of the peduncles. The sections were subsequently stained using safranin O and fast Green (BaiQianDu Biotechnology, Wuhan, China). The anatomical structure of the fruit peduncles was examined under a microscope (Nikon Eclipse E100, Japan) coupled with a Nikon DS-U3 imaging system. Micrographs were also taken using a scanning electron microscope (SEM) (Regulus; Hitachi, Japan).

### 2.3 Broad-targeted metabolomics via ultra-performance liquid chromatography–tandem mass spectrometry analysis

Approximately 100 mg of ground sample powder was extracted with 1 mL of 70% methanol containing 0.1 mg/L lidocaine as an internal standard. The extraction was performed overnight at 4 °C, with intermittent vortexing three times to ensure thorough extraction. Following extraction, the samples were centrifuged at 10,000 g for 10 min, and the supernatant was collected. The supernatant was filtered through a 0.22 μm microporous membrane and stored in vials for subsequent analysis.

Metabolic analysis was performed on a SCIEX ExionLC™ AD system coupled with SCIEX 6500+ QTRAP mass spectrometer (Framingham, MA, USA). UPLC separation was performed using a Waters ACQUITY UPLC HSS T3 C18 column (1.8 µm, 2.1 mm × 100 mm) (Milford, MA, USA). The mobile phase consisted of two components: the aqueous phase, which was ultra-pure water with 0.04% acetic acid, and the organic phase, which was acetonitrile with 0.04% acetic acid. A gradient elution was employed, starting with a mixture of water:acetonitrile at 95:5 (v/v) for 0 min, transitioning to 5:95 (v/v) at 11.0 min, maintaining 5:95 (v/v) from 11.0 to 12.0 min, returning to 95:5 (v/v) at 12.1 min, and holding at 95:5 (v/v) until 15.0 min. The flow rate was set to 0.4 mL/min, and the column temperature was maintained at 40 °C. A 5 μL sample was injected for each analysis. The electrospray ionization (ESI) source temperature was set to 550 °C, with a mass spectrometer voltage of 5500 V. The curtain gas (CUR) pressure was 25 psi, and the collision-activated dissociation (CAD) was high. Authentic standards were purchased from BioBioPha Co., Ltd. (Kunming, China) and Sigma-Aldrich (St. Louis, MO, USA).

### 2.4 Plant hormone analysis via ultra-high performance liquid chromatography–multiple reaction monitoring–tandem mass spectrometry

Approximately 100 mg of ground sample powder was transferred into an Eppendorf tube. A 1000 μL aliquot of extract solvent (acetonitrile-methanol, 1:1, containing an internal standard), pre-cooled to -40 °C, was added. The samples were then homogenized, vortexed for 60 s, and sonicated in an ice-water bath for 10 min. Following this, the samples were incubated at -40 °C for 2 h. After incubation, the samples were centrifuged at 12,000 rpm for 15 min at 4 °C. A total of 900 μL of the supernatant was collected and concentrated using a rotary evaporator at 4 °C. The concentrated samples were reconstituted with 90 μL of a 50% methanol aqueous solution, vortexed for 1 min, sonicated for 120 s, and vortexed again for 1 min. The samples were then centrifuged at 12,000 rpm for 10 min at 4 °C. A 70 μL aliquot of the clear supernatant was transferred to an auto-sampler vial for analysis.

UHPLC separation was performed on an ExionLC™ AD UHPLC System (SCIEX, Framingham, MA, USA), equipped with a UPLC Kinetex C18 column (2.1 mm × 100 mm, 2.6 μm; Phenomenex, Torrance, CA, USA). Mobile phase A consisted of 0.1% formic acid in water, while mobile phase B was 0.1% formic acid in methanol. The column temperature was maintained at 25 °C, while the auto-sampler temperature was set to 4 °C. A 2 μL injection volume was used for each analysis. For mass spectrometry analysis, a 6500 QTRAP+ Mass Spectrometer (SCIEX, Framingham, MA, USA) was employed, equipped with an electrospray ionization (ESI) interface. Ion source parameters were as set as follows: ion spray voltage = ± 4500 V, ion source gas 1 = 50 psi, ion source gas 2 = 50 psi, temperature (+/-) = 450 °C/400 °C, and curtain gas = 40/35 psi.

### 2.5 Transcriptomic profiling using Illumina RNA-sequencing technology

Total RNA was extracted using the Trizol reagent kit (Invitrogen, Carlsbad, CA, USA) and its quality was assessed through the Agilent 2100 Bioanalyzer (Santa Clara, CA, USA) and RNase-free agarose gel electrophoresis. Eukaryotic mRNA was enriched using Oligo(dT) beads, followed by fragmentation of the mRNA into short fragments using the NEBNext Ultra RNA Library Prep Kit for Illumina (NEB, Ipswich, MA, USA), and reverse transcription to synthesize cDNA. The purified double-stranded cDNA underwent end repair, A-tail addition, and Illumina adapter ligation. The adapter-ligation reaction was purified using AMPure XP Beads and size-selected through agarose gel electrophoresis. After PCR amplification, the cDNA library was constructed. Finally, the library was sequenced using the Illumina Novaseq6000 system, with sequencing performed by Gene Denovo Biotechnology Co., Ltd. (Guangzhou, China).

### 2.6 Bioinformatics analysis

Differential expression analysis was performed using the DESeq2 package (Love et al., 2014) in R (v4.4.1), with genes identified as differentially expressed if p-value < 0.05 and variable importance in projection (VIP) > 1. Functional enrichment analysis was conducted using the clusterProfiler package (Yu et al., 2012) in R, in which Gene Ontology (GO) enrichment was performed with the enrichGO function, while KEGG pathway enrichment was conducted using the enrichKEGG function. The significance threshold for all enrichment results was set at p-value < 0.05. Visualization was carried out using ggplot2 (Wickham, 2011) to generate bubble plots. Correlation analysis was conducted using the Gephi software (v0.10.0) (Bastian et al., 2009). Only significantly altered hormone metabolites (VIP > 1.0, p-value < 0.05) and key differentially expressed genes (DEGs) (|log2FC| > 1, p-value < 0.05) were included as network nodes. Edges represented correlations between metabolite-hormone, and metabolite-hormone interactions, with Pearson correlation |r| > 0.6 and p-value < 0.05. Partial least squares discriminant analysis (PLS-DA) was conducted using the ropls package (Thevenot, 2016) in R. Heatmaps were generated with the pheatmap package (Kolde and Kolde, 2015). Venn diagrams were constructed using the VennDiagram package (Chen and Boutros, 2011). Principal component analysis (PCA) was performed and visualized using the ggplot2 package in R.

### 2.7 Quantitative polymerase chain reaction analysis

The extracted RNA was electrophoresed to assess RNA quality. cDNA was synthesized using the TransScript^®^ One-Step gDNA Removal and cDNA Synthesis SuperMix (TransGen Biotech, Beijing, China), and the qPCR system was prepared using a qPCR kit (Polymerase, MF015). qPCR amplification was performed using a real-time fluorescence quantitative PCR instrument (Hehui Biotechnology, Suzhou, China). Relative mRNA expression levels were calculated using the 2^-ΔΔCt^ method, with *EF1α* as the internal reference gene for normalization. Primer sequences are listed in **Table 1**.

**Table 1:** Primer sequences.

Genes	Forward primers (5′→3′)	Reverse primers (5′→3′)
*PP2C51*	GTCAAGGGCTGGTCTTCCTC	TTAATGACCCTTCCACCGGC
*NCED3*	GTCACGGTTCGGGATTTTGC	GCTCCTCCCAAGCATTCCAT
*IAA27*	GAGCTTCTGGGAACTGGGTT	TGGCACTCGTCGTCTGATTC
*TIR1*	GTGTTGGAGAGAGTGGTGGG	GCAGTTTCCGATGAAGACGC
*CESA1*	GGCCTTGACACTGATGGGAA	TCCGGCTTTCTTGTGATGCT
*XTH33*	AGCACCACCAGTACAGCATC	GAAAACTGAAGGGTACGCGC
*EF1α*	GCTGCTGAGATGAACAAGCG	GGTCTCGAACTTCCACAGGG

### 2.8 Statistical analysis

Data were analyzed using RStudio 4.4.1 and Prism 8.0.2 (GraphPad). Comparisons between two groups were performed using an unpaired two-tailed t-test. Each experiment was repeated at least three times, and data are presented as the mean ± standard deviation. A p-value < 0.05 was considered statistically significant.

## 3 Results

### 3.1 Anatomical observations of the AZ in mulberry pedicels

**Figure 1:**
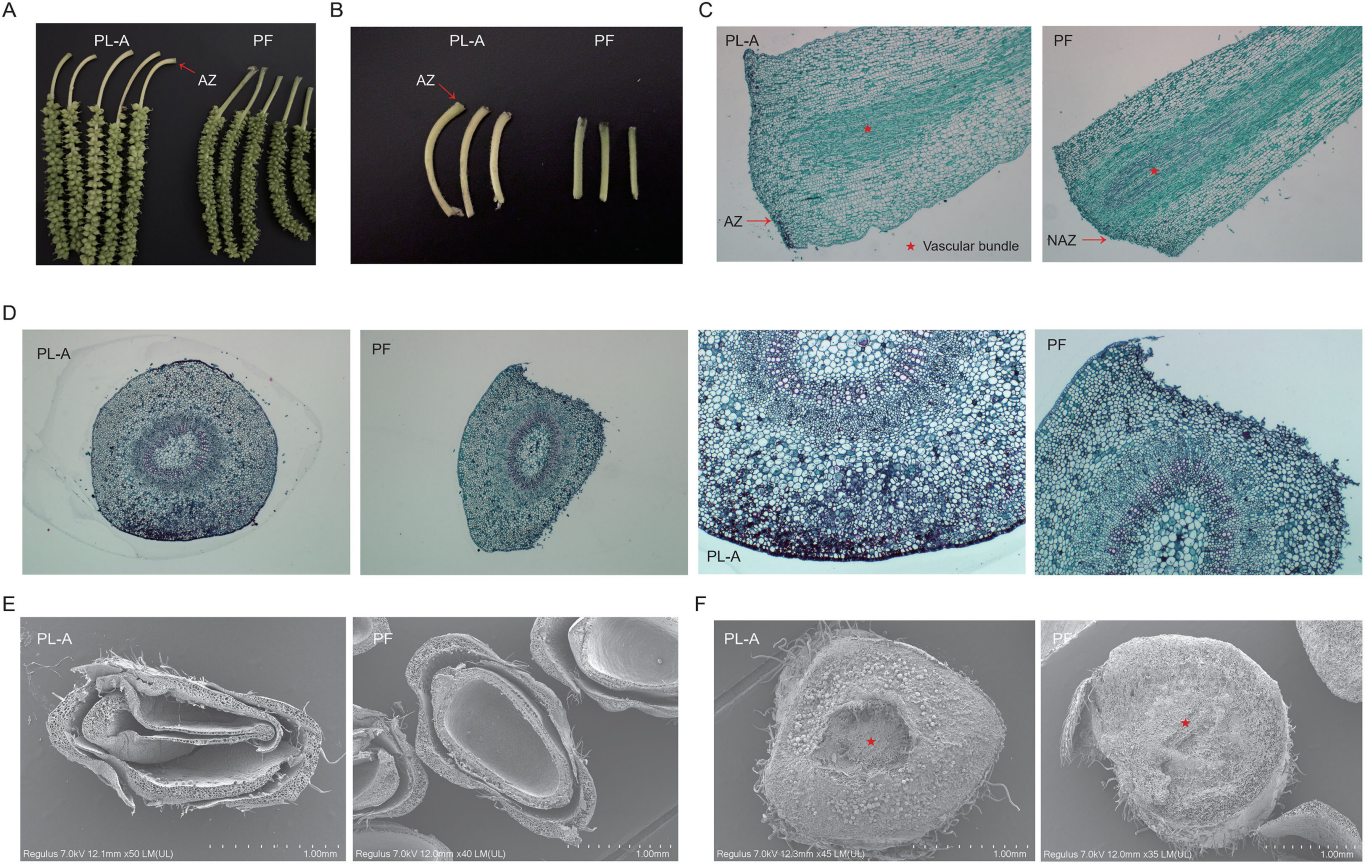
Microscopic structure of longitudinal and transverse sections of peduncles of abscising fruits (PL-A) and non-abscising fruit (PF). **(A)** Representative images of fruit-bearing peduncles from PL-A and PF. **(B)** Representative images of peduncles without attached fruits from PL-A and PF. **(C)** Microscopic structure of the longitudinal section of peduncle from PL-A and PF. AZ: abscission zone; NAZ: non- abscission zone. **(D)** Microscopic structure of the cross-section of peduncles from PL-A and PF. **(E, F)** Representative scanning electron microscope (SEM) images of the abscission zones (AZs) from PL-A and PF. Red stars denote the vascular bundles, and arrows highlight the AZ and NAZ.

To reveal the differences between non-abscising and abscising fruit peduncles in mulberry, both macroscopic appearance and the microstructure of the AZ were observed. Macroscopic observations showed that the inflorescences and peduncles of PL-A samples exhibited elongated morphology and slight yellowing, suggesting potential modified nutrient allocation and hormonal regulation. (**Figures 1A, B**). Comparative histological structure through longitudinal (**Figure 1C**) and transverse (**Figure 1D**) sections revealed disorganized cell arrangement in PL-A peduncles. In contrast, non-abscised peduncles maintained intact cellular organization with tightly packed cells. SEM analysis of transverse sections further highlighted structural features characteristic of the AZ in the PL-A group, including a loose cellular architecture and collapsed vascular bundles (**Figure 1E**). In comparison, the PF group maintained an intact and compact tissue structure (**Figure 1F**). Moreover, cells in the PL-A peduncle were irregularly arranged, with signs of cell wall rupture and partial detachment, in stark contrast to the orderly arrangement and structural integrity of cells observed in the PF peduncle (**Figures 1E, F**). These structural differences suggest that the tissue structure of abscising fruit peduncles was weaker, potentially making the fruit more susceptible to abscission.

### 3.2 Metabolomic overview revealed distinct metabolic profiles among groups

PLS-DA revealed clear separation among the three sample groups, with strong consistency and correlation observed among replicates within each group (**Supplementary Figure S1A**). Heatmap and volcano plot analyses further highlighted substantial differences in metabolic profiles across groups, suggesting distinct metabolic responses (**Supplementary Figure S1B–E**). Differential analysis identified 186, 948, and 126 DAMs between PL-A and PL-N, PL-A and PF, and PL-N and PF, respectively. The greatest metabolic differences were observed between PL-A and PF, with 882 upregulated and 65 downregulated metabolites in PL-A. (**Supplementary Figure S1F**). Furthermore, this comparison group also showed the largest number of uniquely accumulated metabolites (**Supplementary Figure S1G**).

### 3.3 Metabolite enrichment analysis during fruitlet abscission in mulberry

**Figure 2:**
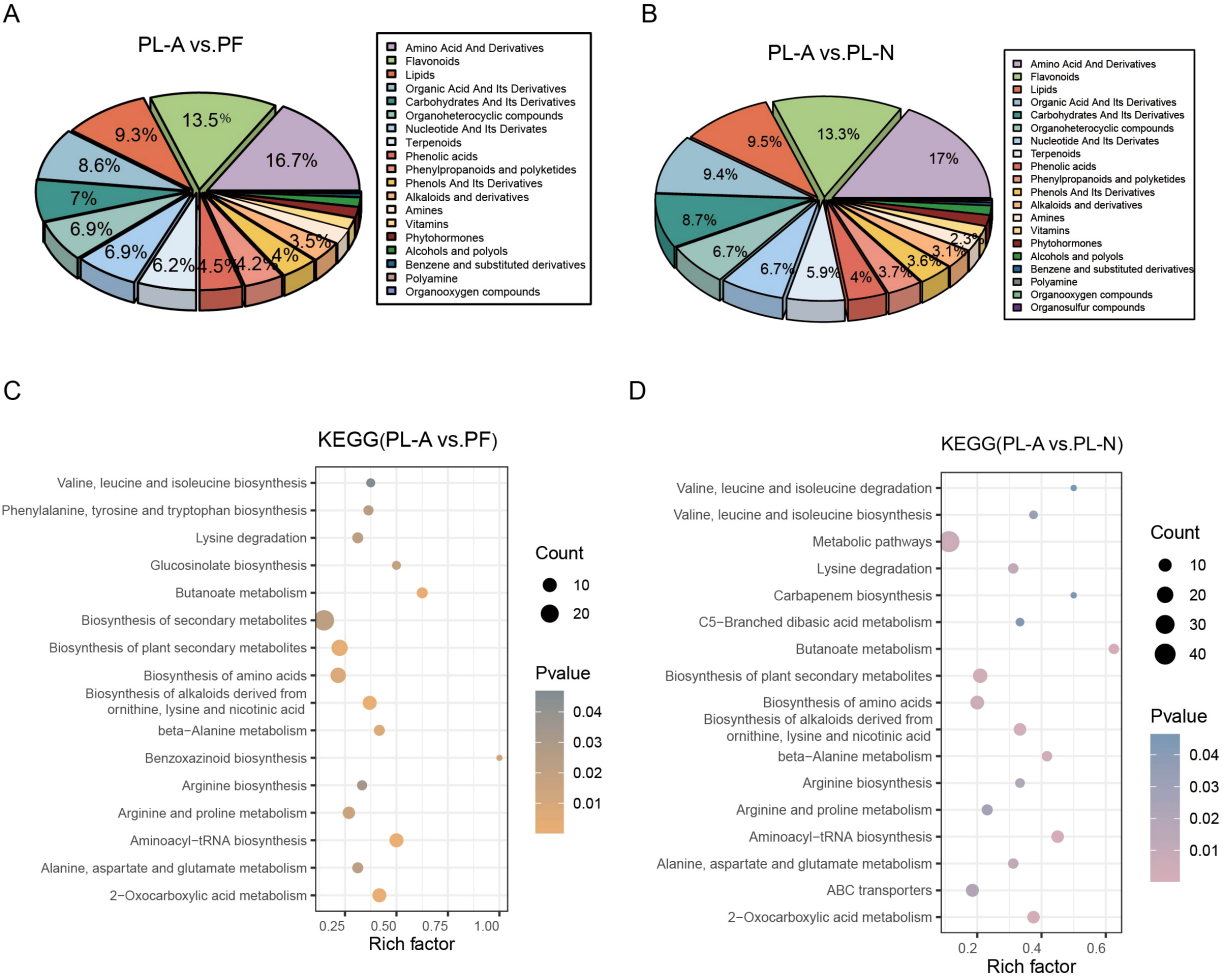
Metabolite enrichment analysis during the fruitlet abscission process in mulberry. **(A, B)** Classification pie charts of DAMs between PL-A and PF **(A)**, and PL-A and PL-N **(B)**. **(C, D)** KEGG pathway enrichment analysis of DAMs between PL-A and PF **(C)**, and PL-A and PL-N **(D)**.

To investigate the metabolic changes during mulberry fruit abscission, we compared the categories and pathway enrichments of DAMs among groups. The results showed that between PL-A and PF, DAMs were mainly enriched in amino acids and derivatives (16.7%), flavonoids (13.5%), lipids (9.3%), and organic acids and derivatives (8.6%) (**Figure 2A**). Similarly, in the comparison between PL-A and PL-N, amino acids and derivatives (17%) and flavonoids (13.3%) also accounted for a large proportion (**Figure 2B**). These may suggest that these two classes of metabolites may play key roles in the fruit abscission process. To further analyze the functions of these DMAs, KEGG pathway enrichment analysis was performed. We found that the DAMs between PL-A and PF, significantly enriched pathways included biosynthesis of plant secondary metabolites, biosynthesis of amino acids, and butanoate metabolism (**Figure 2C**). In the comparison between PL-A and PL-N, in addition to the enrichment of biosynthesis of plant secondary metabolites and biosynthesis of amino acids, pathways such as 2-oxocarboxylic acid metabolism, ABC transporters, and C5-branched dibasic acid metabolism were also significantly enriched (**Figure 2D**). These results further suggest that amino acid metabolism, organic acid metabolism, and substance transport may play key roles in the regulation of fruit abscission.

### 3.4 Hormonal alterations and network connectivity during mulberry fruitlet abscission

**Figure 3:**
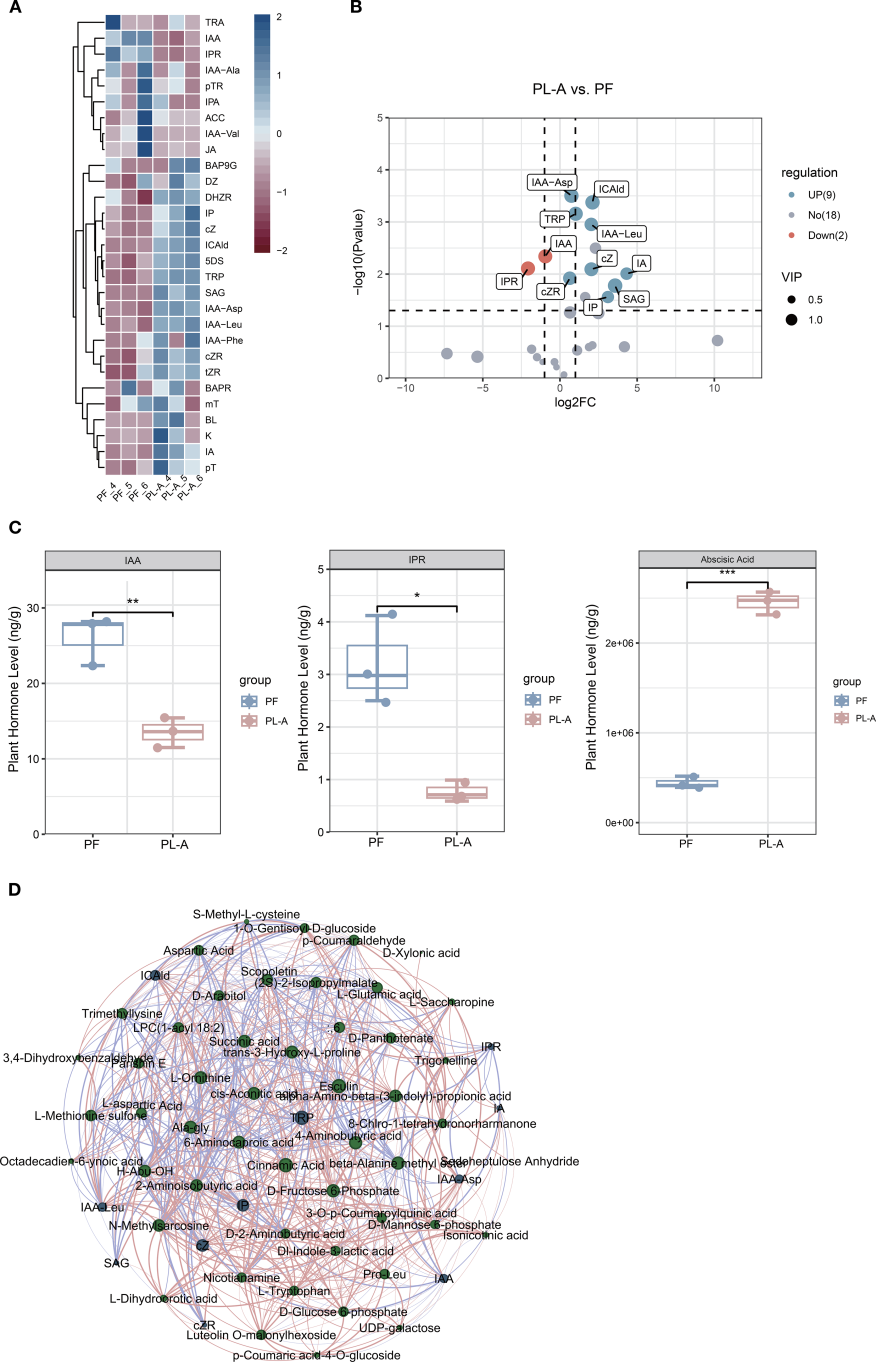
Hormone metabolite analysis in peduncles of PF and PL-A. **(A)** Heatmap showing the abundance of differential hormone metabolites between the PF and PL-A groups. **(B)** Volcano plots displaying differential hormone metabolites between PL-A and PF, p-value < 0.05, VIP > 1. **(C)** Comparison of indole-3-acetic acid (IAA), isopentenyladenosine (IPR), and abscisic acid (ABA) levels between PF and PL-A. **(D)** Correlation network between metabolites and hormones in PL-A and PF. Green nodes represent metabolites and blue nodes represent hormone metabolites. Node size reflects the number of connections (degree), and edge thickness indicates the strength of the correlation.

Plant hormones are crucial regulators in fruitlet abscission, where even subtle fluctuations in hormone levels can initiate complex physiological signaling cascades that determine fruit retention or detachment. To delve deeper into the hormonal basis of mulberry fruit abscission, we performed hormone metabolomics analysis on six fruit peduncle samples (three replicates each from PF and PL-A). In total, 11 hormone metabolites were significantly differentially accumulated during abscission, with 9 upregulated and 2 downregulated (**Figures 3A, B**). Notably, we observed that the levels of IAA and isopentenyladenosine (IPR) in the PL-A group was significantly lower than that in the PF group (P < 0.01), while ABA level was significantly elevated in the PL-A group (P < 0.001) (**Figure 3C**). These results may suggest that auxin depletion and ABA accumulation serve as molecular triggers for fruitlet abscission in mulberry. Further network analysis showed that auxin, cis-zeatin, and tryptophan occupied central positions in the network, indicating their strong connectivity with other metabolites. Specifically, multiple auxin conjugates (e.g., IAA-Ala, IAA-Leu) showed strong associations with other metabolites in the network, suggesting their potential role in fruit abscission (**Figure 3D**). These significant hormonal changes suggest that a hormonal imbalance may occur during the process of mulberry fruitlet abscission.

### 3.5 Transcriptomic overview revealed distinct gene expression patterns among groups

To investigate the molecular mechanisms underlying mulberry fruitlet abscission, nine samples were collected for RNA-seq analysis. PCA results showed clear separation between the three sample groups, with relatively small within-group variation (**Supplementary Figure S2A**). A total of 582 uniquely expressed genes were detected in the PL-A group (**Supplementary Figure S2B**). Minimal transcriptomic changes were observed between PL-N and PF, with 448 upregulated and 776 downregulated DEGs (**Supplementary Figures S2C, F**). In contrast, a total of 3,206 DEGs were identified between PL-A and PF, including 1,446 upregulated and 1,760 downregulated genes (**Supplementary Figures S2D, F**). Similarly, 2,807 DEGs were identified between PL-A and PL-N, comprising 1,130 upregulated and 1,677 downregulated genes (**Supplementary Figures S2E, F**).

### 3.6 KEGG enrichment analysis of DEGs

**Figure 4:**
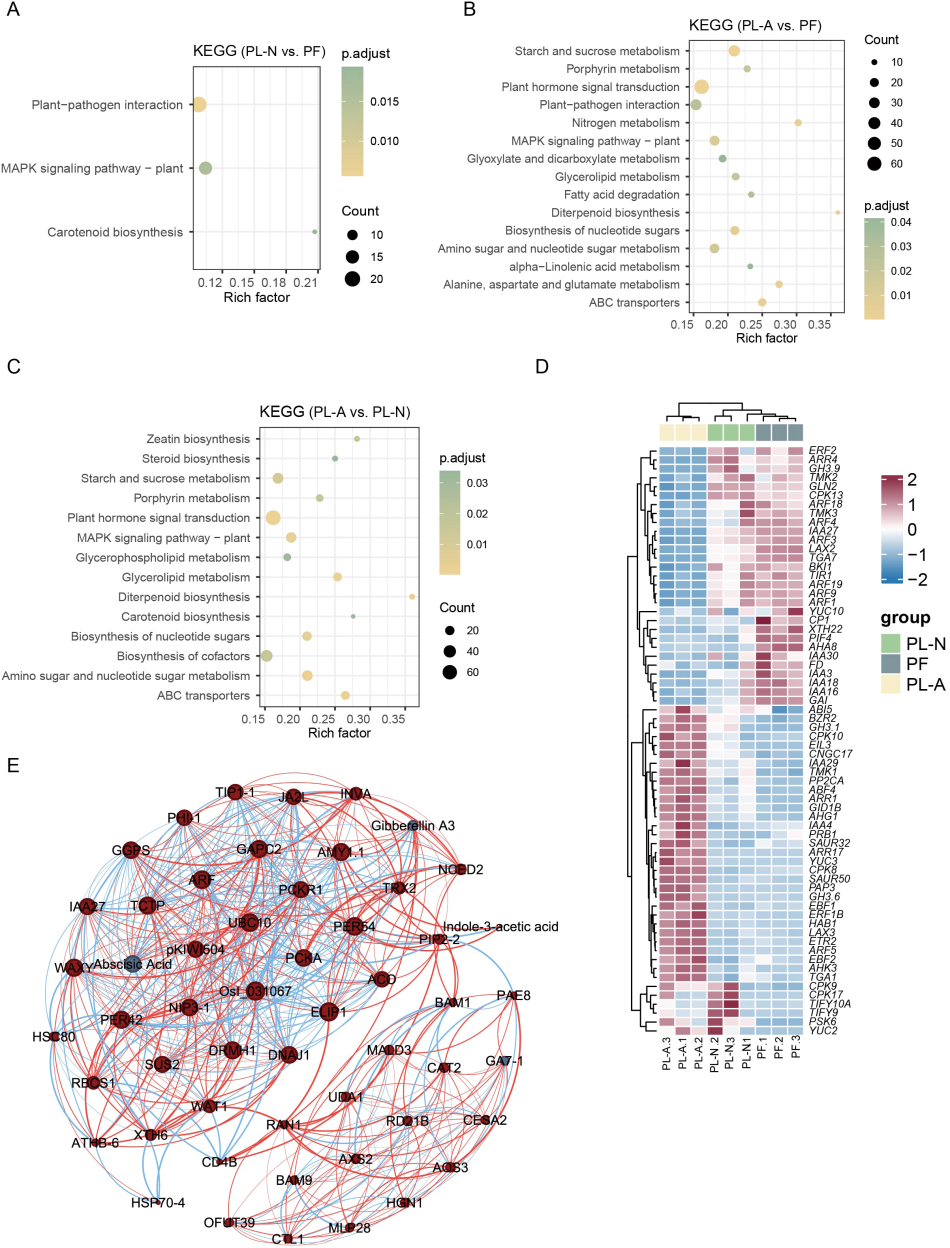
KEGG enrichment analysis of differentially expressed genes (DEGs). **(A–C)** KEGG enrichment analysis of DEGs between PL-N and PF **(A)**, PL-A and PF **(B)**, as well as PL-A and PL-N **(C)**. **(D)** Heatmap showing the expression profiles of genes involved in the plant hormone signal transduction pathway. **(E)** Correlation network between key plant hormones and genes in the PL-A and PF groups. Red lines indicate positive correlations, while blue lines indicate negative correlations. Red dots represent genes, and blue dots represent hormones. Node size corresponds to the number of connections (degree), and edge thickness reflects the strength of the correlation.

To investigate the roles of DEGs in mulberry fruitlet abscission, KEGG pathway enrichment analysis was performed. In the comparison between PL-N and PF, significantly enriched pathways included plant-pathogen interaction, MAPK signaling pathway-plant, and carotenoid biosynthesis, with the first two pathways showing the highest enrichment levels (**Figure 4A**). Between PL-A and PF, as well as PL-A and PL-N, DEGs were primarily enriched in starch and sucrose metabolism, and plant hormone signal transduction pathways (**Figures 4B, C**), indicating their potential involvement in fruit abscission. Heatmap clustering analysis of genes enriched in the plant hormone signal transduction pathway revealed significant differences between PL-A and PF (**Figure 4D**). Furthermore, gene-hormone network was established to explore regulatory relationships between genes and key plant hormones. ABA occupies a central position in the regulatory network, and its interactions with multiple genes highlight its critical role in regulating the fruitlet abscission process (**Figure 4E**). Additionally, hormones such as gibberellin A3 (GA3) and auxin were significantly associated with multiple genes in the network, further suggesting a synergistic regulatory effect of these hormones in the abscission process.

### 3.7 DEGs involved in plant hormones, cell wall biosynthesis, and signal transduction

**Figure 5:**
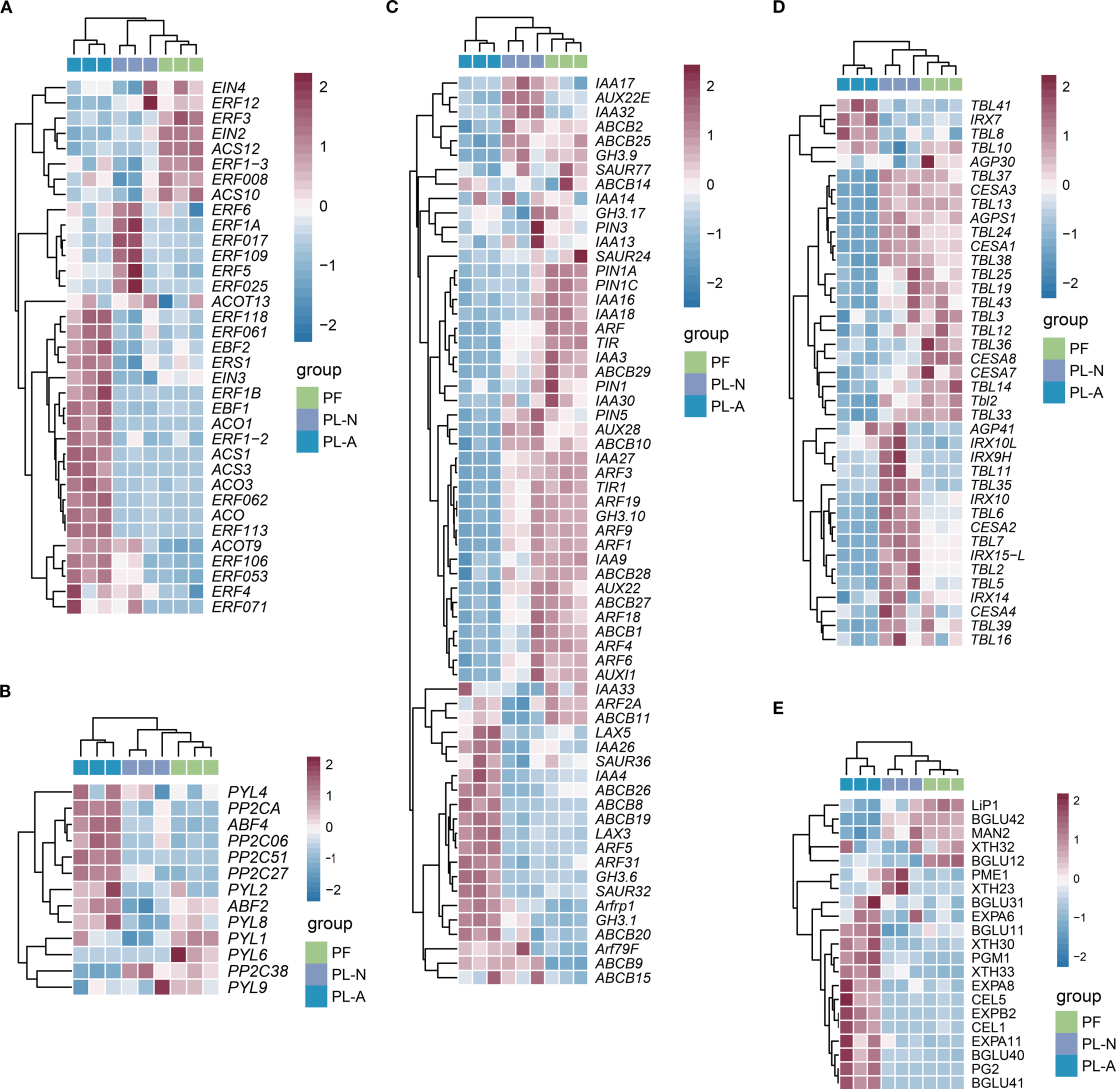
DEGs involved in plant hormones, cell wall biosynthesis, and signal transduction. **(A)** Heatmap of genes involved in ethylene biosynthesis and signal transduction. **(B)** Heatmap of genes involved in ABA biosynthesis and signal transduction. **(C)** Heatmap of genes involved in auxin biosynthesis and signal transduction. **(D)** Heatmap of genes involved in cell wall biosynthesis. **(E)** Heatmap of genes involved in cell wall degradation.

Previous studies have demonstrated the importance of plant hormones in fruit abscission (Yuan et al., 2005). Therefore, we performed a clustering analysis of genes involved in the biosynthesis and signal transduction of ethylene, ABA, and auxin. In the PL-A group, most of the genes related to ethylene (**Figure 5A**) and ABA (**Figure 5B**) were upregulated, while genes related to auxin (**Figure 5C**) were downregulated, which was consistent with the results of the hormone metabolomics analysis. Moreover, in the PL-A group, genes related to cell wall biosynthesis, such as the *TBL* family and *CESA* family, were downregulated, leading to a reduced cell wall synthesis rate and possibly weakening the strength or stability of the cell wall (**Figure 5D**). Meanwhile, the expression of genes related to cell wall-degrading enzymes was upregulated, causing looser cell-to-cell connections, increasing the possibility of fruit abscission (**Figure 5E**).

### 3.8 Starch and sucrose metabolism played essential roles in fruitlet abscission

**Figure 6:**
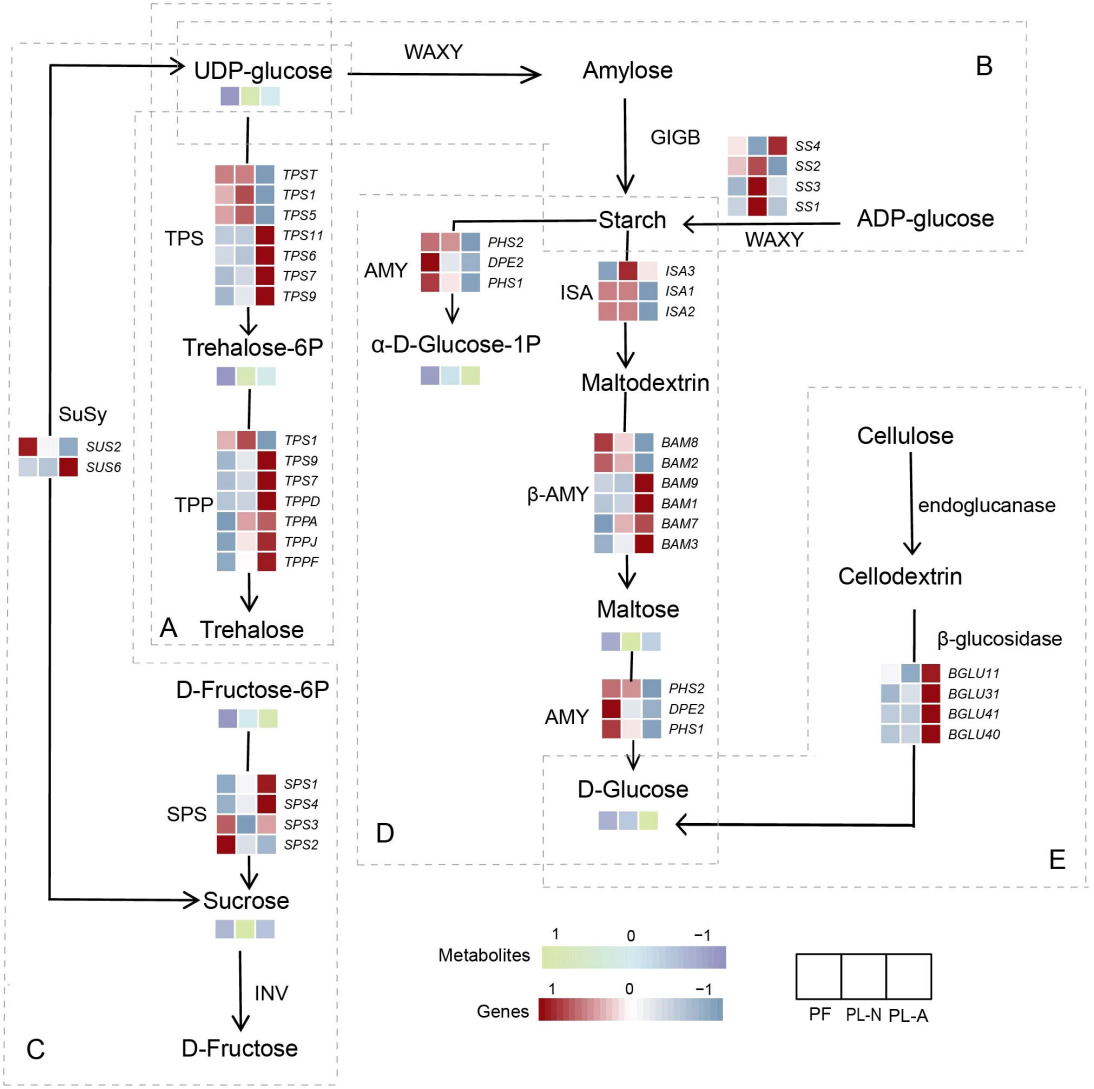
Pathway analysis of gene involved in starch and sucrose metabolism. **(A)** Biosynthesis pathway of trehalose. **(B)** Biosynthesis pathway of starch. **(C)** Biosynthesis and hydrolysis pathway of sucrose. **(D)** Hydrolysis pathway of starch. **(E)** Hydrolysis pathway of cellulose.

Starch and sucrose are the major carbohydrates in plants, playing crucial roles in energy supply, signal transduction, and response to environmental stress (Chen et al., 2023b; Ruan, 2012). Previous studies have reported that starch and sucrose metabolic pathways are important in the fruit abscission process (Agustí et al., 2024). Consistently, our KEGG enrichment analysis revealed significant enrichment of the starch and sucrose metabolism pathway. Notably, genes involved in this pathway exhibited distinct expression patterns between PF and PL-A. In the starch metabolism pathway, the expression of starch-degrading enzymes (such as β-amylase, BAM) was upregulated in the PL-A group, leading to the rapid degradation of starch into maltose and glucose, thus providing energy for abscission process (**Figure 6**). In addition, genes related to cellulose degradation, such as β-glucosidase, were also upregulated in the PL-A group, indicating enhanced cell wall breakdown within the AZ. In the trehalose metabolism pathway, trehalose-6-phosphate phosphatase (TPP) expression was upregulated in the PL-A group, which may be an adaptive response of the plant to environmental stress and resource reallocation. Trehalose, as a protective sugar, helps plants maintain cellular stability under stress conditions. During the fruit abscission process, the plant faces reallocation of energy and carbon sources, and increased expression of TPP helps release trehalose, providing an additional energy source to support plant growth and repair. These changes in gene expression revealed the potential mechanisms by which sucrose and starch metabolism influence fruit abscission (**Figure 6**).

### 3.9 q-PCR validation of genes associated with mulberry fruitlet abscission

**Figure 7:**
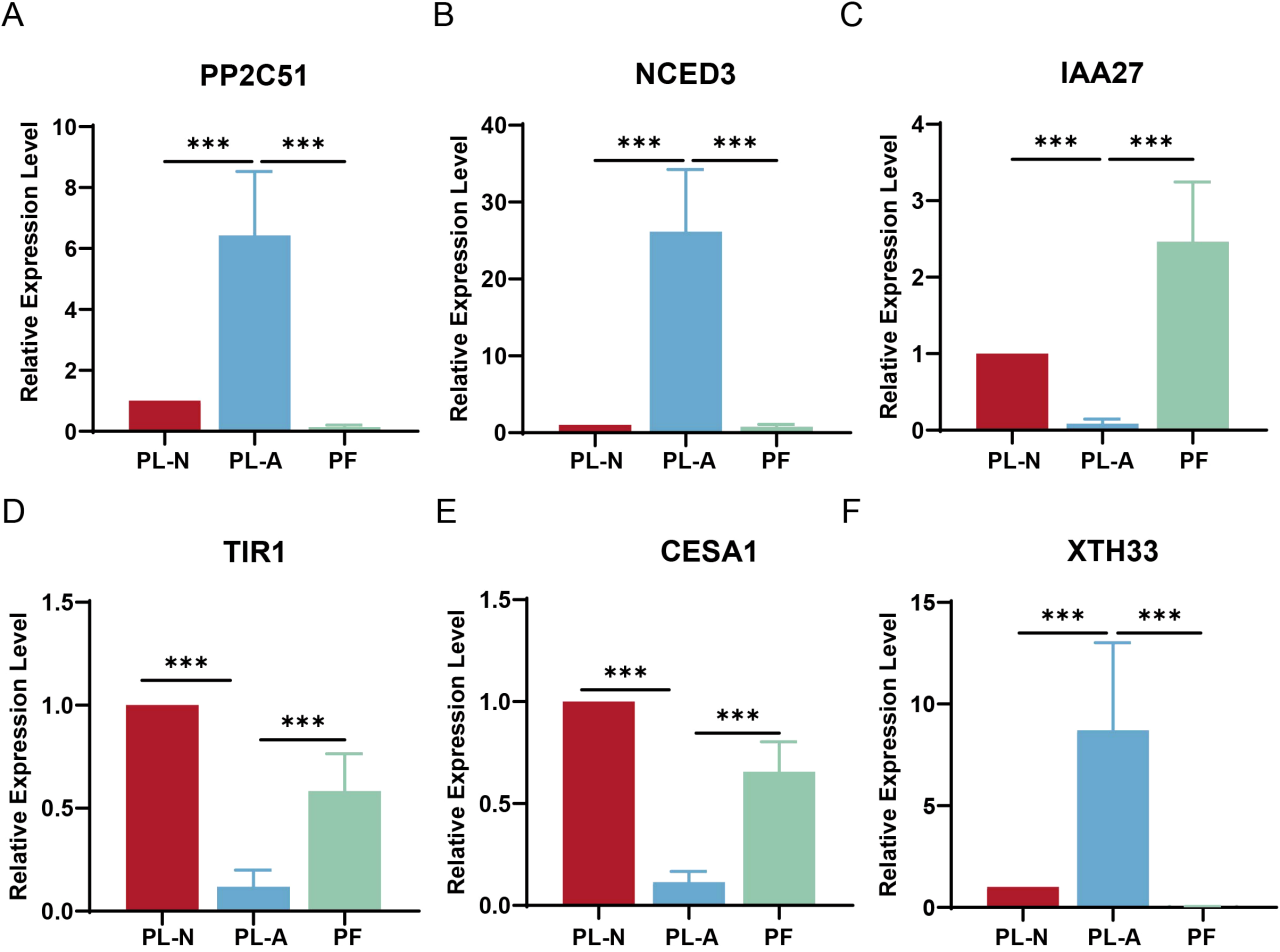
Quantitative polymerase chain reaction (q-PCR) validation of gene expression related to mulberry fruitlet abscission. Relative expression of *PP2C51* **(A)**, *NCED3* **(B)**, *IAA27* **(C)**, *TIR1* **(D)**, *CESA1* **(E)**, and *XTH33* **(F)** in peduncles of PL-A compared with PF. ***P < 0.001 (unpaired two-tailed t-test). Data are presented as mean ± standard deviation.

Based on transcriptomic and hormone metabolomic analyses, ABA, auxin, as well as the synthesis and degradation of the cell wall were closely associated with the abscission of mulberry fruitlets. Therefore, six genes associated with these processes were selected for further analysis: *PP2C51*, *NCED3*, *IAA27*, *TIR1*, *CESA1*, and *XTH33* (**Figure 7**). The results showed that the expression of these genes was consistent with the transcriptomic data. During fruit abscission, the expression of *PP2C51*, *NCED3*, and *XTH33* was significantly upregulated (P < 0.001). Among them, *PP2C51* and *NCED3* are closely related to the synthesis and signal transduction of abscisic acid, while *XTH33* is a gene that promotes cell wall degradation. Their upregulation indicates that increased ABA levels and cell wall loosening are key factors leading to fruitlet abscission. Furthermore, the expression of auxin synthesis and signal transduction-related genes (*IAA27*, *TIR1*) and cell wall synthesis gene (*CESA1*) was significantly downregulated during fruit abscission, suggesting that changes in auxin levels are also an important factor in fruitlet abscission. The q-PCR results further validated that the changes in ABA and auxin content, as well as cell wall degradation, were crucial in mulberry fruitlet abscission.

## 4 Discussion

Mulberry fruitlet abscission is a common physiological phenomenon during fruit development, which significantly affects the yield and quality of mulberries. In-depth research into the mechanisms of mulberry fruitlet abscission is crucial for improving mulberry breeding and cultivation practices. In this study, we found that significant changes in auxin and ABA levels, downregulated expression of genes related to cell wall synthesis, and the enrichment of sucrose and starch metabolic pathways all play crucial roles in regulating fruitlet abscission.

Plant hormones play crucial roles throughout the abscission process, as they mediate the response of plant organs to environmental and developmental stress signals (Estornell et al., 2013; Peleg and Blumwald, 2011; Smékalová et al., 2014). Hormones may either promote or inhibit abscission depending on their concentration, physiological balance, and transport dynamics within plant tissues, reflecting the intricacy of this regulatory network (Sawicki et al., 2015). For example, ethylene, ABA, and jasmonic acid are generally recognized as signals that promote abscission (Brown, 1997; Giulia et al., 2013; Liu et al., 2022), while auxin and GA are considered to inhibit this process (Ben-Cheikh et al., 1997; Yang et al., 2021).

This study showed that during the process of mulberry fruitlet abscission, the significant changes in auxin and ABA levels further highlighted the central role of hormones in regulating fruitlet abscission. The accumulation of ABA in the fruitlet abscission samples significantly increased, and was positively correlated with the high expression of genes related to ABA synthesis, indicating that ABA may promote fruitlet abscission by inducing the formation of the AZ. Meanwhile, auxin plays a dual role in the regulation of fruitlet abscission. It is generally accepted that high auxin levels maintain organ attachment, while a decrease in auxin concentration is necessary to permit abscission. In our study, auxin levels were significantly reduced in the abscissing peduncle, and the expression of auxin biosynthesis-related genes also showed a downward trend. This auxin depletion may enhance the capacity of the AZ to respond to separation signals, thereby facilitating the onset of fruitlet abscission in mulberry.

Cell wall degradation is a key physiological mechanism in fruit abscission, as the structural integrity of cell wall in the AZ is directly related to the strength of attachment between the fruit and the parent plant (Merelo et al., 2017). Transcriptomic analysis in this study revealed that the expression of genes encoding cell wall-modifying enzymes, such as cellulase (*CEL5* and *CEL1*) and expansin (*EXPA6*, *EXPA8*, and *EXPA11*), was upregulated in the abscissing samples, indicating that these enzymes play a key role in fruitlet abscission. During fruitlet abscission, the upregulation of cellulase expression leads to the degradation of cellulose, gradually breaking down and softening the cell wall in the fruit, weakening the connection between the fruit and the plant, and ultimately resulting in abscission (Jiang et al., 2019). The elevated expression of cell wall-degrading enzymes in the AZ has also been widely reported in other fruit abscission studies. For example, in blueberries and lychees, the activation of these enzymes represents a crucial step in the abscission process (Chen et al., 2023a). Genes encoding cellulases, pectinases, PGs, expansin, xyloglucan endotransglucosylases/hydrolases, and peroxidases were found to be significantly upregulated during the abscission cycle in litchi (Li et al., 2015) and tomato (Nakano et al., 2012). Additionally, the degradation of the cell wall may be a result of hormonal regulation. Both ABA and ethylene can promote the expression of cell wall-degrading enzymes, thereby accelerating the formation of the AZ (Jiang et al., 2019; Wei et al., 2010). Therefore, the upregulation of cell wall-degrading enzymes during mulberry fruitlet abscission may be a combined effect of ABA accumulation and ethylene signaling activation. This phenomenon further supports the diversity of plant hormones in regulating fruitlet abscission.

Metabolomic and transcriptomic analyses in this study revealed that sucrose and starch metabolism pathways play a crucial role in the mulberry fruitlet abscission process. The research shows that the energy supply to the fruit is closely related to its growth, development, and abscission, with sucrose and starch metabolism providing the necessary carbon sources and energy for the fruit (Zhang et al., 2022). The significant enrichment of sucrose and starch metabolism pathways likely provided energy to the cells to meet the demands for cell wall degradation and other metabolic processes associated with the abscission process. In normally developing fruits, sucrose metabolism provides the energy required for growth, whereas during abscission, alterations in sucrose transport and degradation may affect carbon allocation and weaken fruit attachment. Therefore, sucrose level is considered an important factor in triggering the fruitlet abscission process, as demonstrated in *Citrus unshiu* (Gómez-Cadenas et al., 2000; Mehouachi et al., 1995). Additionally, higher levels of ABA can inhibit starch synthesis, thereby affecting fruit growth and development (Zhu et al., 2011a). In summary, this study suggests that changes in energy metabolism pathways may support carbohydrate redistribution to fuel cell wall degradation and fruit abscission.

## 5 Conclusion

This study explored the molecular mechanisms and pathways involved in mulberry fruitlet abscission. Through physiological experiments, metabolomics, and transcriptomics, we observed differences between the peduncles of non-abscising and abscising mulberry fruits. The abscising samples displayed typical AZ characteristics, including large intercellular spaces and loose cellular architecture. Multi-omics research revealed a complex molecular regulatory process involving numerous genes and metabolites. These findings provide valuable insights into the molecular mechanisms and pathways of mulberry fruitlet abscission and may contribute to the development of more effective breeding strategies. Further research is needed on the biosynthesis and signaling of auxin and ABA, as well as the regulatory genes controlling major components of the cell wall during the fruitlet abscission process.

## Data Availability

The data presented in this study have been deposited in the NCBI Sequence Read Archive (SRA) under BioProject accession number PRJNA1327559.
