# Supplementary material for: Unraveling the mechanisms of fruit abscission in Morus laevigata through multi-omics approaches
Source: Front Plant Sci. 2025 Sep 22;16:1605312. doi: 10.3389/fpls.2025.1605312 (PMC12498018; doi:10.3389/fpls.2025.1605312)
Supplement: Supplementary file 1 [file DataSheet1.docx]

**Supplementary Table 1 Number of Fruit Retained at Different Days after Flowering**

| **YT Variety** | | | | | | | | | |
| --- | --- | --- | --- | --- | --- | --- | --- | --- | --- |
| Days after flowering | 1.1 | 1.2 | 1.3 | 2.1 | 2.2 | 2.3 | 3.1 | 3.2 | 3.3 |
| 10 days | 92 | 80 | 66 | 90 | 101 | 94 | 86 | 100 | 104 |
| 15 days | 88 | 78 | 61 | 87 | 101 | 92 | 86 | 98 | 101 |
| 20 days | 42 | 34 | 32 | 57 | 61 | 52 | 56 | 76 | 73 |
| 25 days | 37 | 29 | 31 | 48 | 49 | 51 | 54 | 75 | 67 |

| **MT Variety** | | | | | | | | | |
| --- | --- | --- | --- | --- | --- | --- | --- | --- | --- |
| Days after flowering | 1.1 | 1.2 | 1.3 | 2.1 | 2.2 | 2.3 | 3.1 | 3.2 | 3.3 |
| 10 days | 118 | 142 | 143 | 185 | 146 | 152 | 187 | 141 | 190 |
| 15 days | 118 | 141 | 141 | 184 | 144 | 151 | 186 | 141 | 185 |
| 20 days | 114 | 141 | 140 | 178 | 143 | 148 | 186 | 139 | 184 |
| 25 days | 113 | 141 | 140 | 177 | 142 | 146 | 186 | 138 | 184 |

Each code (e.g., 1.1, 2.3) represents a specific tree and its branch. The number before the decimal indicates the tree number, and the number after the decimal indicates the branch number. Fruit drop rate is calculated as the proportion of fruits dropped relative to the initial count:

Fruit Drop Rate (%) = (Initial Fruit Number-Remaining Fruit Number)/ Initial Fruit Number ×100


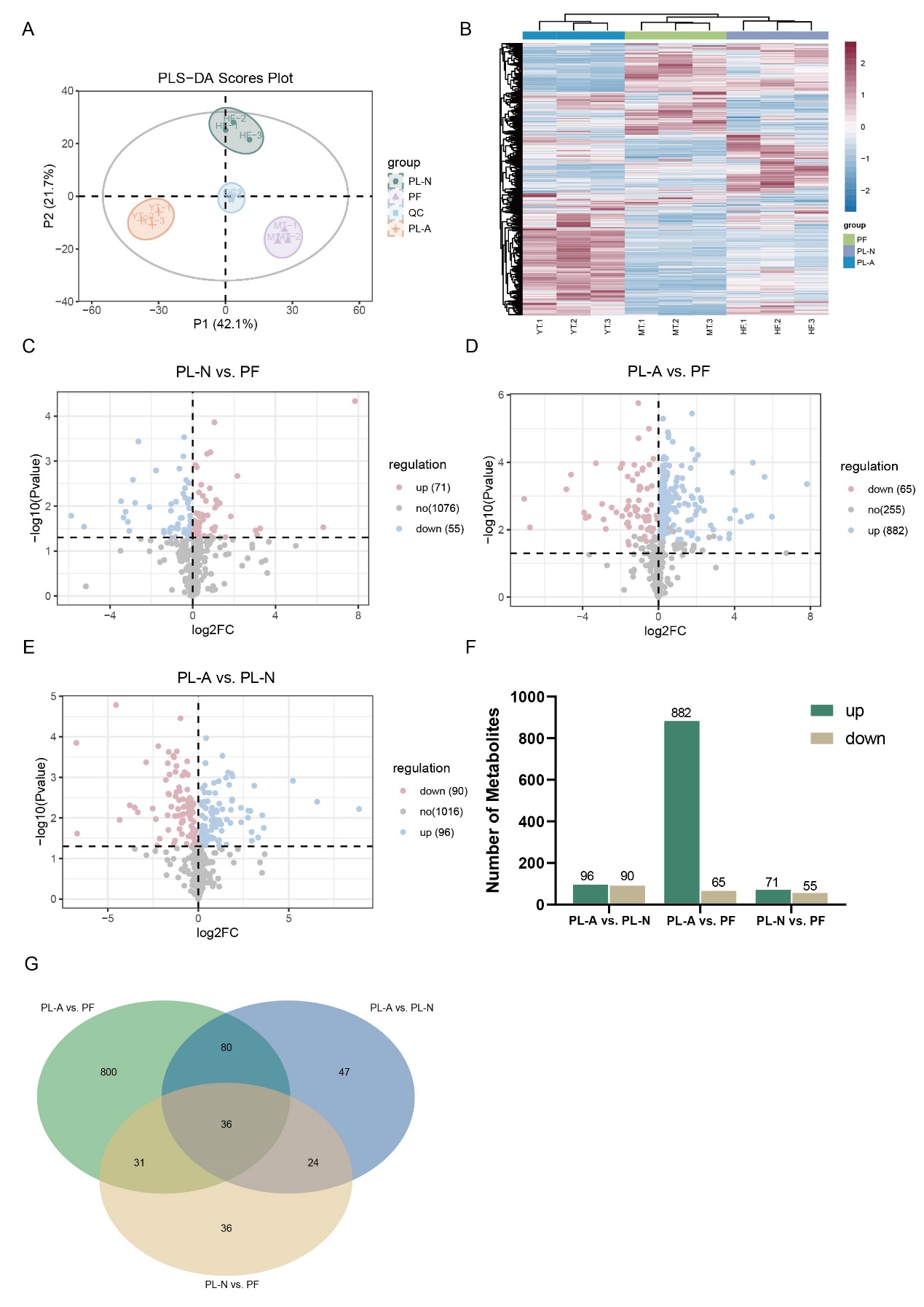


**Figure S1**. Metabolomic overview revealed distinct metabolic profiles among the three groups. **(A)** Partial least squares discriminant analysis (PLS-DA) of mtabolomic profiles. Each point represents an individual replicate. **(B)** Heatmap showing the expression patterns of all detected metabolites across the three sample groups. **(C-E)** Volcano plots displaying differentially accumulated metabolites (DAMs) between each pairwise comparison (p-value < 0.05, VIP > 1, where VIP refers to Variable Importance in Projection, indicating the contribution of each variable to the PLS-DA model). **(F)** Bar chart summarizing the number of upregulated and downregulated metabolites in each pairwise comparison. **(G)** Venn diagram illustrating the number of unique and shared DAMs among comparison groups.


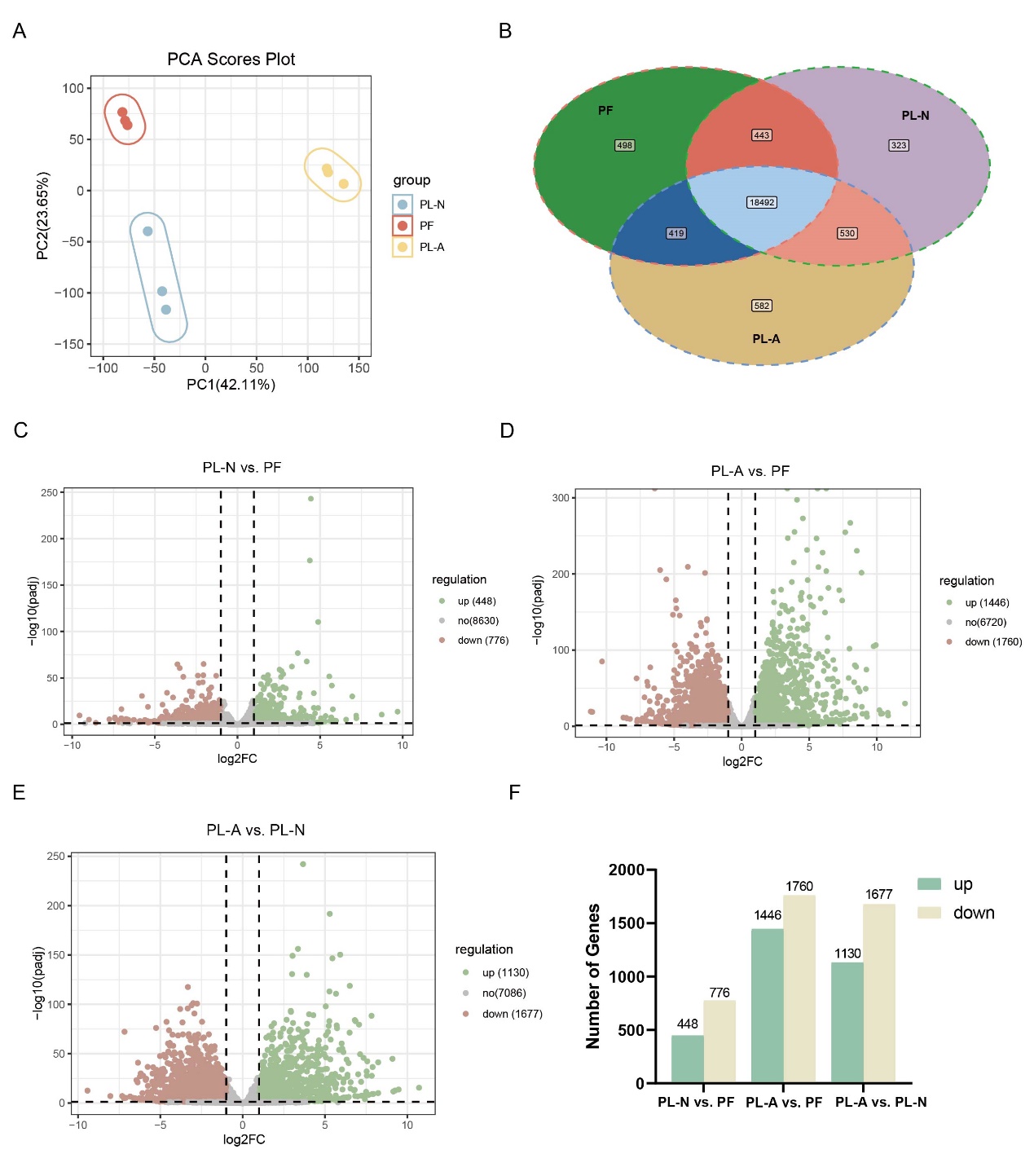


**Figure S2**. Transcriptomic overview revealed distinct gene expression patterns among groups. **(A)** PCA of transcriptome data. **(B)** Venn diagram showing the number of uniquely and shared expressed genes between groups. **(C-E)** Expression of differentially expressed genes (DEGs) between PL-N and PF (**C**), PL-A and PF (**D**), as well as PL-A and PL-N (**E**). (|log2FC|>1, P <0.05). **(F)** Bar chart showing the number of DEG between groups.
